# Supplementary material for: Association of Genomic Instability Score, Tumor Mutational Burden, and Tumor-Infiltrating Lymphocytes as Biomarkers in Uterine Serous Carcinoma
Source: Cancers (Basel). 2023 Jan 15;15(2):528. doi: 10.3390/cancers15020528 (PMC9856872; doi:10.3390/cancers15020528)
Supplement: Supplementary file 1 [file cancers-15-00528-s001.zip › cancers-1998680-supplementary.pdf]

Supplementary Materials

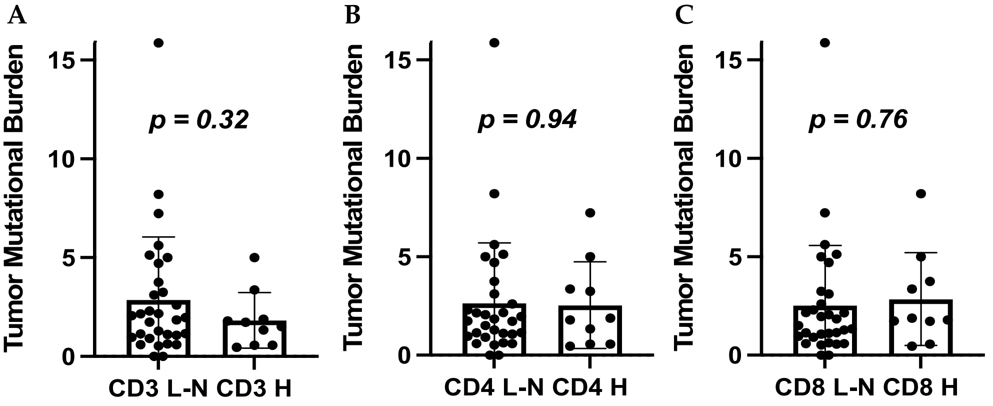

Figure S1. Comparison of mean tumor mutational burden score by high (H) and low-normal (L-N) immune cell counts. **A**: CD3, **B**: CD4, **C**: CD8.

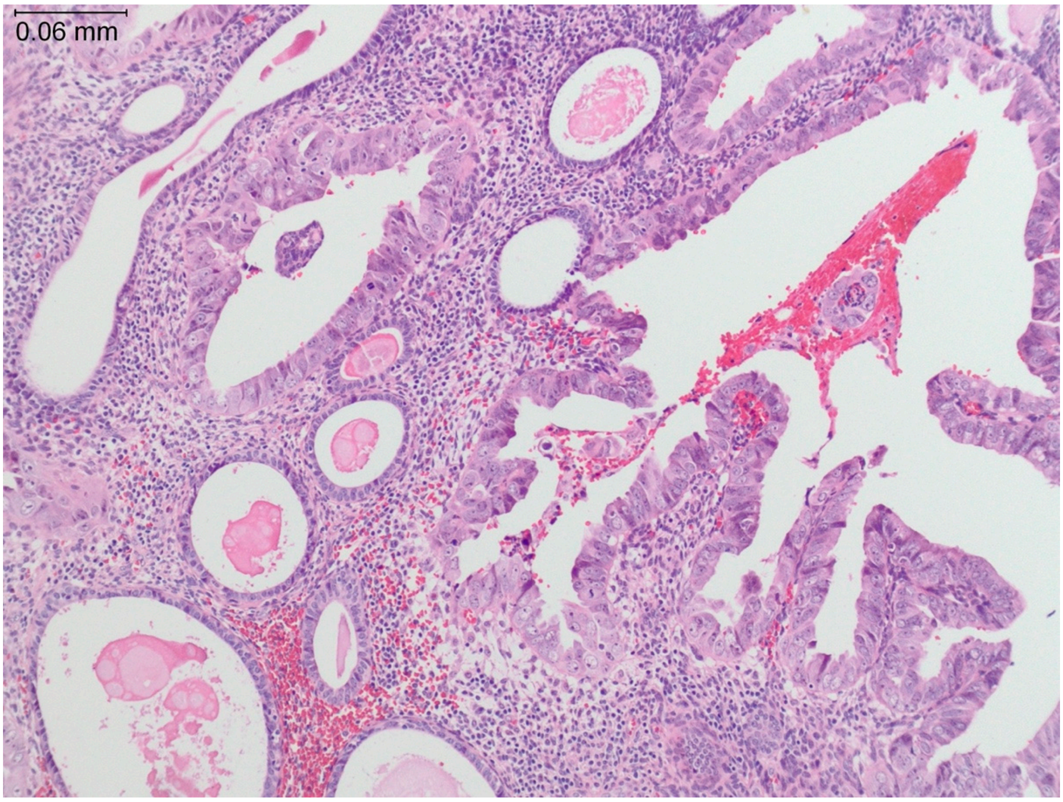

Figure S2. H&E of the tissue from the patient with a tumor containing a mutation in *MSH6*.

Table S1. Mutations identified in genes involved in the homologous recombination pathway in this sample of uterine serous tumors. Stratified by HRD score (>31, ≤31, and failed to calculate).

		TP53		BRCA1	BRCA2	ATM		BARD1	BRIP1
GIS score	ID	Suspected Pathogenic	Unknown Pathogenicity	Unknown Pathogenicity	Unknown Pathogenicity	Deleterious or Suspected Deleterious	Unknown Pathogenicity	Unknown Pathogenicity	Unknown Pathogenicity
GIS ≥ 31	308165	c.855_856delinsTT (p.Glu285_Glu286delinsAsp*)							
	314624	c.659A>G (p.Tyr220Cys)							
	308171	c.568_572del (p.Pro190Serfs*17)							
	308161	c.722C>G (p.Ser241Cys)							
	308167	c.733G>T (p.Gly245Cys) c.817C>T (p.Arg273Cys)							
	314617	c.581T>G (p.Leu194Arg)						Uncharacterized Large Rearrangement	
	308153	c.818G>T (p.Arg273Leu)							
	308172	c.715A>G (p.Asn239Asp)							
	314625	c.843C>A (p.Asp281Glu)							
	314632		c.763A>T (p.Ile255Phe)						
	308160		c.451C>G (p.Pro151Ala)						
	314619	c.1025del (p.Arg342Glnfs*3)							
	314639	c.830G>T (p.Cys277Phe)		dup exon 24					
	308163	c.742C>T (p.Arg248Trp)							
	308158	c.532C>G (p.His178Asp)							
	308169	c.524G>A (p.Arg175His)							
	314637		c.839G>C (p.Arg280Thr)						
	314634	c.637C>T (p.Arg213*)							
	314644					c.6443_6452+19del	c.646G>T (p.Ala216Ser) c.968T>A (p.Ile323Lys)		c.1735C>T (p.Arg579Cys)
	314641								
GIS < 31	308164	c.517G>A (p.Val173Met)							
	314621	c.818G>A (p.Arg273His)							
	314631	c.841G>T (p.Asp281Tyr)					c.2927T>C (p.Val976Ala)		
	308159		c.737T>C (p.Met246Thr)						
	308170	c.371_372del (p.Cys124Tyrfs*24)			dup exons 1-2				
	314623	c.659A>G (p.Tyr220Cys)							
	314628	c.733G>A (p.Gly245Ser)							
	308173	c.818G>A (p.Arg273His) c.843C>A (p.Asp281Glu)							
	314640	c.396G>C (p.Lys132Asn) c.673-1G>T							
	314618	c.713G>T (p.Cys238Phe)							
	308156	c.1034del (p.Asn345Metfs*25)			c.2573G>C (p.Arg858Thr) c.3097G>C (p.Asp1033His)				
	308166		c.622_623delinsAT (p.Asp208delinsIle)						
	314627	c.637C>T (p.Arg213*)	c.322G>A (p.Gly108Ser) c.472C>T (p.Arg158Cys)						
	308155	c.380C>T (p.Ser127Phe)							
	314620	c.73del (p.Leu25Tyrfs*19)	c.773A>G (p.Glu258Gly)						
	308168	c.743G>A (p.Arg248Gln) c.836G>A (p.Gly279Glu)							
	314633	c.526T>C (p.Cys176Arg)							
	314638					c.1564_1565del (p.Glu522Ilefs*43)	c.8683G>C (p.Glu2895Gln)		
	308162						c.6347+5G>A		
	308174								
Failed	308154	c.743G>A (p.Arg248Gln)							
	308157	c.437G>A (p.Trp146*)							
	308176		c.503A>G (p.His168Arg)						
	314616	c.488A>G (p.Tyr163Cys)							
	314622	c.722C>T (p.Ser241Phe)							c.778A>G (p.Thr260Ala)
	314626	c.818G>A (p.Arg273His)							
	314629	c.742C>T (p.Arg248Trp)							
	314630	c.467G>C (p.Arg156Pro)							
	314636	c.830G>T (p.Cys277Phe)							
	314642		c.775G>T (p.Asp259Tyr)						
	314635								
	314643								

		CHEK1	CHEK2	FAM175A	MRE11A	NPN	PALB2	RAD51C	RAD51D
GIS score	ID	Unknown	Unknown	Unknown	Unknown	Unknown	Unknown	Unknown	Unknown
GIS ≥ 31	308165								
	314624						c.2587-6T>G		
	308171				c.315-4dupT				
	308161								
	308167								
	314617								
	308153								
	308172				c.1002C>G (p.Ser334Arg)				
	314625				c.2092A>G (p.Met698Val) c.315-4dupT				
	314632		c.1198G>T (p.Gly400Trp)					c.1085C>T (p.Ser362Phe)	
	308160								
	314619								
	314639								
	308163								
	308158				Uncharacterized Large Rearrangement				
	308169								
	314637								
	314634								
	314644				c.37T>A (p.Phe13Ile)	c.1317A>G (p.Ile439Met)			
	314641	c.467G>A (p.Arg156Gln)			c.121G>A (p.Asp41Asn)	c.1354A>C (p.Thr452Pro)			
GIS < 31	308164					c.1317A>G (p.Ile439Met)			
	314621				c.315-4dupT				
	314631				c.2092A>G (p.Met698Val) c.315-4dupT				
	308159				c.315-4dupT				
	308170								
	314623								
	314628			c.917T>C (p.Val306Ala)					
	308173				c.315-4dupT				
	314640								
	314618								
	308156								
	308166								c.26G>C (p.Cys9Ser)
	314627								
	308155								
	314620				c.1783+5G>C c.2092A>G (p.Met698Val)				
	308168								
	314633								
	314638								
	308162								
	308174					c.1845+3A>G			
Failed	308154						c.1072C>T (p.Pro358Ser)		
	308157								
	308176								
	314616								
	314622						c.2756T>C (p.Val919Ala)		
	314626								
	314629								
	314630				c.315-4dupT				
	314636								
	314642								
	314635								
	314643								
